# Hypothesized pathogenesis of acardius acephalus, acormus, amorphus, anceps, acardiac edema, single umbilical artery, and pump twin risk prediction

**DOI:** 10.1002/bdr2.1976

**Published:** 2021-12-20

**Authors:** Martin J. C. van Gemert, Michael G. Ross, Jeroen P. H. M. van den Wijngaard, Peter G. J. Nikkels

**Affiliations:** ^1^ Department of Biomedical Engineering & Physics Amsterdam UMC Amsterdam The Netherlands; ^2^ Department of Obstetrics and Gynecology Harbor UCLA Medical Center Torrance California USA; ^3^ Department of Clinical Chemistry, Hematology and Immunology Diakonessenhuis Utrecht Zeist Doorn Utrecht The Netherlands; ^4^ Department of Pathology, Wilhelmina Children’s Hospital University Medical Center Utrecht The Netherlands

**Keywords:** acardiac monochorionic twins, acardiac morphology, Hyrtl's anastomosis, short umbilical cord syndrome, spontaneous aborted embryos

## Abstract

**Background:**

Acardiac twinning complicates monochorionic twin pregnancies in ≈2.6%, in which arterioarterial (AA) and venovenous placental anastomoses cause a reverse circulation between prepump and preacardiac embryos and cessation of cardiac function in the preacardiac. Literature suggested four acardiac body morphologies in which select (groups of) organs fail to develop, deteriorate, or become abnormal: acephalus (≈64%, [almost] no head, part of body, legs), amorphus (≈22%, amorphous tissue lump), anceps (≈10%, cranial bones, well‐developed), and acormus (≈4%, head only). We sought to develop hypotheses that could explain acardiac pathogenesis, its progression, and develop methods for clinical testing.

**Methods:**

We used qualitatively described pathophysiology during development, including twin‐specific AA and Hyrtl's anastomoses, the short umbilical cord syndrome, high capillary permeability, properties of spontaneous aborted embryos, and Pump/Acardiac umbilical venous diameter (UVD) ratios.

**Results:**

We propose that each body morphology has a specific pathophysiologic pathway. An acephalus acardius may be larger than an anceps, verifiable from UVD ratio measurements. A single umbilical artery develops when one artery, unconnected to the AA, vanishes due to flow reduction by Hyrtl's anastomotic resistance. Acardiac edema may result from acardiac body hypoxemia combined with physiological high fetal capillary permeability, high interstitial compliance and low albumin synthesis. Morphological changes may occur after acardiac onset. Pump twin risk follows from UVD ratios.

**Conclusion:**

Our suggested outcomes agree reasonably well with reported onset, incidence, and progression of acardiac morphologies. Guidance for clinical prediction and testing requires ultrasound anatomy/circulation study, from the first trimester onward.

## 1 INTRODUCTION

Monozygotic (identical), monochorionic (a single placenta), diamniotic (two amniotic sacs) twin pregnancies require that embryonic splitting of the zygote occurs between 3 and 8 days following fertilization. The pregnancy is monoamniotic (one amniotic sac) when the splitting is between 8 and 13 days. Conjoint twins develop when the splitting is between 13 and 16 days (fig. 2 of Benirschke & Kim, 1973). These pregnancies can have a number of potentially serious complications because placental anastomoses connect the circulations of both twins in ≈98% of cases (Nikkels, Hack, & van Gemert, 2008). These anastomoses can be superficial, only chorionic vessels, or deep. Superficial anastomoses are arterioarterial (AA) and less frequently venovenous (VV), with a flow that can be bidirectional. Deep anastomoses are arteriovenous (AV) and venoarterial (VA), which occur at the capillary level within a placental cotyledon, where the chorionic arterial inflow is from one twin and the chorionic venous outflow to the other twin.

Acardiac twinning is a rare complication of monochorionic twins, which was first reported by Benedetti (1533) (see the historical overview by Obladen, 2010). Acardiac twinning develops in about 2.6% of monochorionic twins and, depending upon frequency and method of assisted reproduction, happens about once per 9,500–12,000 pregnancies (van Gemert, van den Wijngaard, & Vandenbussche, 2015). Acardiac onset occurs in the first trimester only (van Gemert, Ross, van den Wijngaard, & Nikkels, 2021; van Gemert, van der Geld, Ross, Nikkels, & van den Wijngaard, 2021), and requires a set of AA and VV anastomoses. It was demonstrated by ultrasound that during acardiac development a reverse flow in the arteries and aorta occurs (hence the name twin reverse arterial perfusion [TRAP] sequence; Van Allen, Smith, & Shepard, 1983). This results in the cessation of cardiac function in the future acardiac embryo. The other twin, the pump twin, then perfuses the acardiac body by its arterial blood through reverse flow via the AA and return back to the pump twin through the VV. Notably, the pump twin arterial flow is poorly oxygenated compared to venous blood, as it does not undergo placental gas exchange. Thus, the AA and VV anastomoses function to both create cardiac cessation and to permit growth of the acardiac body. Frequently, acardiac twins are edematous and have one umbilical artery. Natural pump twin mortality, commonly a result of cardiac failure, is close to 40% (Table 1). A general accepted and clinically applied method for effective risk prediction of pump twin complications is still lacking, despite recent work that proposed a simple clinical approach based on Pump/Acardiac umbilical venous diameter (UVD) ratios (Section 3.8 and Appendix A).

**TABLE 1:** Pump twin mortality reported in literature

Study	Total cases	Demised
Chi (1989)	8	3
Moore, Gale, and Benirschke (1990)	49	27
Healey (1994)	117	41
Søgaard, Skibsted, and Brocks (1999)	6	5
Brassard, Fouron, Leduc, Grignon, and Proulx (1999)	9	4
Shih et al. (1999)	5	2
Dashe, Fernandez, and Twickler (2001)	6	1
Sullivan, Varner, Ball, Jackson, and Silver (2003)	10	1
Chanoufi et al. (2004)	6	5
Lewi, Valencia, Gonzalez, Deprest, and Nicolaides (2010)	24	8
Hartge and Weichert (2012)	6	0
Overall	246	97/246(≈39%)

*Note*: The first nine cases are from Sebire, Wong, and Sepulveda (2006), with a 41% mortality.

The general pathogenesis of acardiac twinning has now been well established (van Gemert, Ross, et al., 2021). Briefly, a future acardiac embryo must have a lower mean arterial pressure than its future pump embryo, either from unequal weights (Benirschke, 2009, from uneven embryonic splitting), or from an abnormality, for example, chromosomal defects (Moore et al., 1987; Søgaard et al., 1999; Van Allen et al., 1983), or congenital anomaly (personal experience PGJN). We observed an 11 weeks old monochorionic twin case with a normal pump twin and an abnormal acardiac twin with multiple congenital anomalies, that is, holoprosencephaly and unilateral renal agenesis (case presented during the 62nd Pediatric Pathology Society Annual Meeting, 2016, Lund, Sweden). Sufficiently larger mean arterial pressures in the future pump than the future acardiac embryos cause an arterial perfusion from future pump to future acardiac via the AA, which becomes a retrograde perfusion flow into the aorta, opposite to the normal directed larger aorta flow. These two opposite flows thus reduces the normal directed aorta flow, and thus also the placental flow, which causes a slowly lowering future acardiac mean arterial pressure. Most likely, a slowly decreasing mean arterial pressure will slowly increase the left ventricular afterload of the future acardiac embryo. The physiological reactions of the developing heart are not comparable with an adult heart due to the not yet developed intercalated discs in the fetal heart (Vreeker et al., 2014). This physiological absence of normal intercalated discs might trigger cardiac arrhythmias and cardiac failure and might increase retrograde flow even further. This deteriorating process, when of sufficient magnitude, continues to impair the cardiac output of the future acardiac embryo until cardiac arrest occurs, which marks the onset of acardiac twinning. An alternative mechanism for acardiac development is spontaneous early demise of the future acardiac embryo, which results in the reverse flow via the AA anastomosis and return flow to the pump via the VV.

Previous literature from the early 20th century on, thoroughly described the anatomy of acardiac fetuses and four body morphologies were suggested. These four body morphologies have widely varying incidences (Table 2). Neither morphology nor incidence can be derived from the general pathogenesis of acardiac twin formation as discussed above. Such derivation requires knowledge of preacardiac/pump embryo pathophysiology, as well as organ development and degeneration under conditions of hypoxemia, which is the normal situation in the first trimester. Unfortunately, such information is currently not available, which precludes the formulation of a general method that predicts preacardiac behavior, the moment of acardiac onset, its morphology and incidence, and its possible progression after onset.

**TABLE 2:** Incidence and description of the four acardiac morphologies

Acardius	Incidence^a^	Description^b^
Acephalus	152/239 ≈ 64%	Is the most common species. Head wanting, or very rudimentary. Intestines and abdominal organs rudimentary, and the organs above the diaphragm represented by the merest trace. Rarely, more than a few vertebræ present, and these are usually of the lumbar or sacral type. Shoulder girdle undeveloped. Pelvis and lower extremities more nearly perfect. Little more than a lump of connective tissue, covered by edematous skin. Sometimes a tract of hairy scalp is seen. Usually, some attempt at budding out of limbs, though seldom with any systematic bony structure. There may be some rudiments of visceral tissue. Head alone present, but never fully developed
Mylacephalus^c^		An amorphous mass with some development of one or more limbs
Amorphus	53/239 ≈ 22%	Least developed. Little more than a lump of connective tissue, covered by edematous skin. Sometimes a tract of hairy scalp is seen. Usually some attempt at budding out of limbs, though seldom with any systematic bony structure. There may be some rudiments of visceral tissue
Anceps	25/239 ≈ 10%	Is the least atrophied form, characterized by absence or nondevelopment of face, the extreme anterior part of the body. There are rudiments of cranial bones and of the brain. It has more or less perfect trunk and extremities. It presents the transition to well‐developed twins from one ovum. The species is rare From Latin: 2‐headed, uncertain
Acormus	9/239 ≈ 4%	Rarest. Head alone present, but never fully developed From Greek: κορμός (kormós), trunk, body

^a^ Based on 151 cases of “*heartless monsters*” from Napolitani and Schreiber (1960), and 88 cases from Sato, Kaneko, Konuma, Sato, and Tamada (1984).

^b^ Das (1902).

^c^ Related fifth morphology (mentioned by Das, 1902; Simmonds & Gowen, 1925).

Therefore, our first aim was to generate hypotheses that could explain/predict the specific pathophysiologic pathway of each of the acardiac morphologies and their umbilical arterial circulation, based on combining available clinical and scientific information of acardiac twin pregnancies. Our second aim was to suggest methods for clinical prediction and testing of these hypotheses.

## 2 METHODS

We used qualitatively described pathophysiology. We included normal but also abnormal organogenesis, and organ degeneration; twin‐specific AA and Hyrtl's anastomoses; anatomy and circulation of a just formed acardiac twin from a normal embryo; the short umbilical cord syndrome; physiological high capillary permeability, high fetal interstitial compliance, and low‐to‐absent fetal liver albumin synthesis; severe hypoxia‐induced vascular/endothelial damage; embryo versus fetal acardiac/pump weight ratios; biochemical and physical properties of spontaneous aborted embryos, and UVD ratios.

Some of these will be elaborated below, the others will be part of Section 3.

### 2.1 Estimated incidence and morphology of spontaneous aborted monochorionic twin embryos with a normal live cotwin

It can be hypothesized that an acardiac twin may result from the spontaneous demise of a monochorionic twin embryo due to a chromosomal abnormality or congenital defect. Unfortunately, and to the best of our knowledge, no information exists on the frequency and morphology variation of aborted monochorionic embryos with a surviving normal coembryo. In an attempt to provide a first estimate of such statistics, we made the assumption that frequency and morphology of malformations in singleton spontaneous aborted embryos may be representative for this monochorionic twin abortion statistics. Support for this hypothesis comes from Júnior, Martins Santana, and Cecchino (2015), stating that “*The prevalence of congenital anomalies is almost twice when comparing monochorionic twins with dichorionic, although in both cases only one fetus is affected in 90% of the time*.” Such anomalies are usually major (Rustico et al., 2018). We thus hypothesized that these affected monochorionic embryos may have approximately singleton anomalies, and may be affected by similar miscarrying mechanisms, so they will approximately develop as aborted singleton embryos. Obviously, perfusion by retrograde low‐oxygenated arterial blood only occurs in the monochorionic embryos.

**FIGURE 1:**
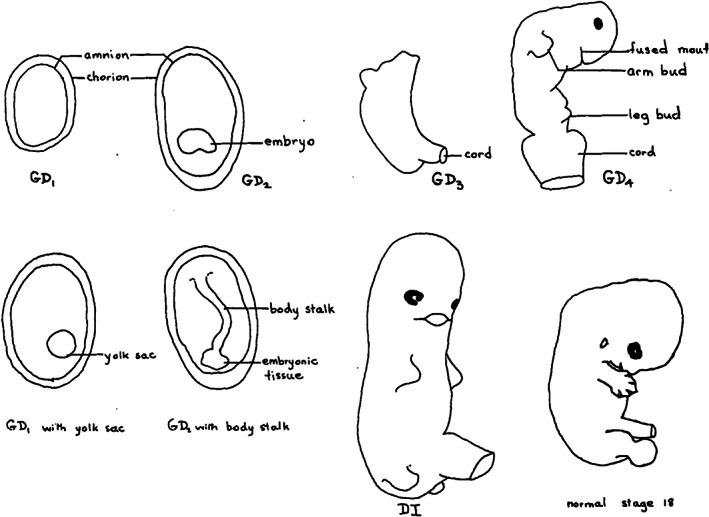
Spontaneous aborted singleton embryos (fig. 1 of Poland et al. (1981), reproduced with permission of the publisher). DI, developmentally inconsistent; GD, growth disorganized. Poland gave 337 GD1, 286 GD2 (1–4 mm; <6 weeks gestation) (179 direct; 107 with body stalk up to 3 cm), 133 GD3 (up to 1 cm; <7 weeks), 31 GD4 (3–17 mm; <8.5 weeks; CRL, 50% <1 cm; <7 weeks), 78 DI (most CRL 1–2.1 cm; 7–9 weeks), 183 normal embryos. In Table 3 we used that 1.6 time more abnormalities occurred in monozygotic twin pregnancies (Myrianthopoulos, 1976), thus assumed also in monochorionic twins, than in singletons, hence we reduced the 183 aborted normal embryos to 183/1.6 ≈ 115. We also included 36 defects of specific systems (DSS) cases (Table 3). Thus, an assumed total of 1,016 spontaneous aborted monochorionic twin embryos with live cotwins

The most extensive and detailed series is by Poland, Miller, Harris, and Livingston (1981) who categorized 1,126 spontaneous aborted singleton embryos into four classes of growth disorganization (GD1–GD4), one class of developmental inconsistency (DI), one class of defects of specific systems (DSS), and normal embryos (Table 3 and Figure 1). Furthermore, table 2 of Myrianthopoulos (1976) showed that in 90 malformed monozygotic twin embryos, about 24.1% malformations occurred compared to 15.6% in 8,288 singletons and 14.8% in 91 dizygotic twins, thus about 1.6 times more in monozygotic than dizygotic and/or singleton pregnancies. We will use this information, for example, in correcting the number of aborted normal embryos from 183 (for singletons, table 1 of Poland et al., 1981) to an estimated 183/1.6 ≈ 115 for aborted normal monochorionic twin embryos. Also, we used a short umbilical cord incidence of 1.6 times the 6% value found in singletons (Naeye, 1985), or 9.6%. In addition, Myrianthopoulos (1976) also provided arguments that DSS aborted monozygotic embryos may cause pregnancy abortion in a significant number of cases. From his table 3, 40 of the 90 monochorionic twin fetuses had major malformations and 9 had “major and minor” malformations, thus 49 with some form of major malformations. We assumed that the corresponding twin pairs could unlikely have survived. Thus, supposing these observations to be representative for monochorionic twin embryos, we keep only 41/90 ≈ 46% of the DSS embryos with a live and normal coembryo, or ≈36 instead of the 78 found in singletons by Poland et al. (1981). We finally included 337 GD1, 179 GD2 embryos directly attached to the amnion and 107 via a body stalk, 133 GD3, 31 GD4, 78 DI, 36 DSS, and 115 normal embryos, or an estimated total of 1,016 spontaneously aborted monochorionic twin embryos with a live normal cotwin (Table 3 and Figure 1).

**TABLE 3:** Growth disorganized (GD), developmentally inconsistent (DI), and defects of specific systems (DSS) spontaneous embryo abortion morphologies (from Poland et al., 1981)

GD1	An intact chorionic sac or amniotic sac with no evidence of embryo or body stalk. A yolk sac may or may not have been present. There were 337 specimens with GD1
GD2	A piece of embryonic tissue 1–4 mm long with no recognizable external features and in which the caudal and cephalic poles could not be distinguished, attached to the amnion either directly (179 specimens) or by a body stalk of up to 30 mm in length (107 specimens)
GD3	A grossly disorganized embryo up to 10 mm in length showing some recognizable morphological features such as retinal pigment and in which the cephalic and caudal poles could be distinguished. There were 133 specimens with GD3
GD4	An embryo with a major distortion of body shape, always including the head. The head was often small. There were 31 specimens with GD4
DI	A group of 78 embryos less disorganized than GD4 but for which the developmental markers were out of step with each other
DSS	A group of 78 embryos with neural tube, eye–ear–neck and musculoskeletal abnormalities. Monozygotic twin pregnancies (Myrianthopoulos, 1976) reduced the 78 by Poland et al. (1981) to 36
Normal	A group of 183 normal embryos, using 1.6 more abnormalities in monozygotic twin pregnancies than singletons (Myrianthopoulos, 1976) reduced the 183 by Poland et al. (1981) to 115

### 2.2 Short umbilical cord syndrome

We will include the hypothesis that a very short umbilical cord of the future acardiac embryo is a novel etiology of acardiac twinning (Section 3.3). Thus, we also hypothesized that the short umbilical cord syndrome could be relevant here because it is associated with severe body defects, such as structural defects, limb/body wall defects, and evisceration of abdominal organs (e.g., Miller, Graham, Higginbottom, & Smith, 1981). Importantly, the reported concordance between the sides of major limb and body wall defects of the upper as well as the lower body side, could facilitate mechanisms of acardius acormus and acardius amorphus formation when the embryos are concomitantly perfused retrograde with low‐oxygenated blood.

## 3 RESULTS

### 3.1 Acardius acephalus and acardius anceps

The formation of these two acardii may differ in their time of onset because the cranium develops around 10 weeks (e.g., Jin, Sim, & Sang‐Dae Kim, 2016), so we hypothesized that acardius acephalus onset is earlier than 10 weeks and acardius anceps after 10 weeks, although we acknowledge that degeneration of already developed organs may occur too, so the separation of onset of these two acardiac forms may not be that strict (see Section 4.1, para. 2). Otherwise, their formation is expected to have many similarities, which frequently shows from their similar body shapes, particularly the common presence of lower extremities. Therefore, we discuss them both in this paragraph.

We first evaluated whether the assumed spontaneous monochorionic embryo abortion data of Table 3 can predict the approximate total incidence of acardius acephalus (64%) and acardius anceps (10%) of Table 2. We assumed (Table 3) that the number of aborted embryos that are expected to include lower extremities could possibly develop into acephalus or anceps morphology, thus the 115 normal and 36 DSS embryos. Their incidence of 151/1,016 ≈ 15% is however significantly lower than the 74% total incidence of these two acephalus and anceps acardiac forms (Table 2). Thus, aborted monochorionic embryo statistics cannot by itself predict the total incidence of these acardiacs, and unequal mean arterial pressures in two live monochorionic embryos must be the major contributing mechanism here. As stated before (Section 1, para. 4), insufficient information is available to predict the incidence of these mechanisms of unequal arterial pressures for acardiac onset.

We found two cases where acardiac onset was actually witnessed. First, Coulam (1996) observed two embryos at 5 and 6 weeks, of which one had lost cardiac activity at 7 weeks, and was diagnosed as an acardiac twin at 11 weeks. There was no development above the thorax. Thus, this acardius acephalus developed between 6 and 7 weeks, indeed before 10 weeks as we hypothesized. Second, Wesley et al. (2011) observed two beating hearts at 6 + 5 weeks and diagnosed TRAP at 8 + 5 weeks. Autopsy (30 + 4 weeks) showed a small bowel without stomach and liver, a small chest cavity, hypoplastic vessels of head and neck, and no cardiac tissue. There were large caliber AA and VV anastomoses between the shared cord insertions. Thus, again an acardius acephalus with confirmation of onset <10 weeks, and including a large AA caliber.

Yamoah, Kotlinska, Coady, and Lesny (2009) identified a demised twin at 8 + 2 weeks, which became an acephalus that included all limbs. The crown rump lengths (CRL) were 20 and 16 mm of pump and acardiac twins. Vasconcelos, Falé Rosado, Torres, Teresa Martins, and Cohen (2017), identified an anceps acardiac twin at 10 + 5 weeks (their fig. 1), Meyberg and Groß (2002) at 10 + 6 weeks, and Stiller, Romero, Pace, and Hobbins (1989) at ≈11 weeks, with 41 mm (pump) and 27 mm (acardiac) CRLs. In our opinion, these observations give support to our onset estimates of acephalus <10 weeks, and anceps >10 weeks.

We further hypothesized that the AA anastomosis has a larger diameter, thus lower resistance, in acephalus than anceps acardiacs, needed to produce the larger preacardiac retrograde perfusion flow for earlier acephalus onset. Interestingly, first trimester acardiac perfusion and growth is controlled by the AA retrograde flow (van Gemert, Ross, et al., 2021, p. 693), suggesting stronger growth of acephalus than anceps acardii. If true, acardiac/pump CRL and UVD ratios are larger for acephalus than anceps. Such observations must be done in the first trimester, without being affected by possible morphology changes after onset (Section 3.6), and subcutaneous acardiac edema (Section 3.5). Here, the two cases that we found, from Yamoah et al. (2009) for acephalus and Stiller et al. (1989) for anceps, confirm our hypothesis, with CRL ratios of 16/20 = 0.8 for acephalus, exceeding the 27/41 ≈ 0.66 for anceps. However, two cases are obviously insufficient for making any statistical relevant conclusion.

### 3.2 Acardius amorphus

We assumed that these acardiacs can be formed from very early retrograde perfusion of low flow, from small embryos with a higher body resistance and likely the presence of a larger diameter AA, which causes serious early hypoxia and severely disturbed development resulting in absence of head, limbs and internal organs. The amorphous acardiac then contains skin, connective tissue and perhaps some central bone fragments. Van Gaever, Defoort, and Dhont (2008) reported such a case at 11 + 4 weeks. No information on the AA anastomosis was given. Also Sharbaf et al. (2008) described this type of acardius amorphus, at 26 weeks, again without AA information.

For an estimate of incidence we have to rely on the assumed incidence of amorphous spontaneous aborted monochorionic twin embryos here (Table 3). The 107 GD2 without body stalk, and the 133 GD3, assumed to include the amorphous specimens that resemble an acardius amorphus, Figure 1, is 240/1,061 ≈ 23%, close to the ≈22% mentioned in Table 2. Nevertheless, we submit that GD3 shapes may well include other, nonamorphus morphologies, thus acardius amorphus formation may also include embryos with unequal mean arterial pressures and very early low flow retrograde arterial perfusion. The incidence of this latter mechanism is however unknown.

### 3.3 Acardiac development related to the short umbilical cord syndrome

When we sought for mechanisms that could explain acardius acormus formation, thus an acardiac twin with a head only, we were left with few options. Neither normal acardiac formation pathogenesis (Section 1, para. 3), nor spontaneous abortion data were applicable, the latter because no acormus form was mentioned by Poland et al. (1981) and Philipp, Philipp, Reiner, Beer, and Kalousek (2003). We then considered the possibility that an acardius acormus can only form if the preacardiac embryo is already prone to malformation of lower extremities and body wall. The short umbilical cord syndrome (Miller et al., 1981) might then be an interesting candidate.

We hypothesized that acardius acormus formation requires that one of the two embryos has a short umbilical cord, a congenital anomaly most likely due to a ventral body wall defect (Vermeij‐Keers, Hartwig, & van der Werff, 1996), that it develops defects associated with this cord abnormality, and becomes retrograde perfused by low‐oxygenated blood during the physiological presence of an omphalocele, at 6–10 weeks. Onset is after 10 weeks, when the skull has been formed. We tacitly assumed spontaneous abortion of the pregnancy when both monochorionic embryos would have a short umbilical cord.

Acormus acardii require a head with a skull, hence GD4, DI, DSS, and normal spontaneous aborted monochorionic twin embryos apply, or a total of (31 + 78 + 36 + 115)/1,016 = 260/1,016 ≈ 25.6% of such aborted cases. Assuming here a short umbilical cord incidence of 1.6 times 6%, or 9.6%, gives an incidence of about 2.5% acormus acardii from aborted embryos, not too far (≈60%) from the 4% of Table 2. It is obvious that live normal coembryos with a very short umbilical cord also apply here.

A literature assessment supports the short umbilical cord hypothesis. Figure 1 of Laure‐Kamionowska, Maślińska, Deręgowski, Oiekarski, and Raczkowska (2004) shows an acormus with a very short umbilical cord. The case by Morizane, Ohara, Mori, and Murao (2002) had a short umbilical cord. Finally, the case of Brand and Krol (1941) most likely included also a short umbilical cord, based on their reported short umbilical cord of the pump twin combined with their fig. 13, showing the acardiac to have an even shorter cord.

Obviously, acardiac twinning as complication of the short umbilical cord syndrome also occurs before 10 weeks. For example, we identified seven acephalus and amorphus literature cases with a short umbilical cord (Table 4), likely formed *before* skull formation, prior to 10 weeks. An interesting example is from Sharbaf et al. (2008), where acardiac formation included loss of a hypoplastic leg and an only remaining intestine with little fluid filled amniotic sac and short umbilical cord. Evisceration of abdominal organs, here an intestinal sac with fluid, is one of the complications of the short umbilical cord syndrome (Miller et al., 1981). We hypothesized that earlier acardiac onset with a short umbilical cord relates to a larger diameter AA and UVD ratio, so a larger retrograde body perfusion during the physiological presence of an omphalocele, at 6–10 weeks.

**TABLE 4:** Amorphus and acephalus acardiac cases with short umbilical cord

Author	Umbilical cord^a^ length/NL/GA	Morphology	Comment
French, Bieber, Bing, and Genest (1998)	6.5/60/42	Amorphus	230 g; dichorionic placenta
Emery, Vaux, Pretorius, Masliah, and Benirschke (2004)	4/53/33	Mylacephalus^b^	Omphalocele, 2 legs, 1 rudimentary arm
Lattanzi et al. (2006)	6/52/32	Amorphus	33 × 40 cm^2^, 1,070 g globular skin mass with hairs
Hanley, Boyd, and Hecht (2007)	6/55/35.6	Amorphus	8.5 × 8.5 × 3.5 cm^3^
Chen, Wu, Chen, and Yang (2007)	2/33/≈20	Acephalus	Reddish pelvic area, 2 legs
Sharbaf et al. (2008)	4/59/38	Acephalus	Intestinal sac with fluid, 1 leg vanished (34 weeks)
Bhat, Butale, Kumbhalkar, and Raut (2017)	10/59/38	Acephalus	Amorphous body of 25 × 10 × 8 cm^3^

^a^ Length (cm); NL, normal length for GA (cm); GA, gestational age (weeks).

^b^ Table 2.

Thus, acardiac twins that develop in combination with the short umbilical cord syndrome create a novel etiology of acardiac onset. Because skull formation is not a necessity here, the total incidence also includes the 133 GD3 specimen, thus creating an additional 133/1,016 ≈ 13.1% candidates for acardiac formation. With 9.6% short umbilical cords thus an additional 1.3% predicted incidence of nonacormus short umbilical cord related acardii. Again, live normal coembryos with a very short umbilical cord also apply here too.

### 3.4 Acardiac vascular anatomy, circulation, and development of organs

**FIGURE 2:**
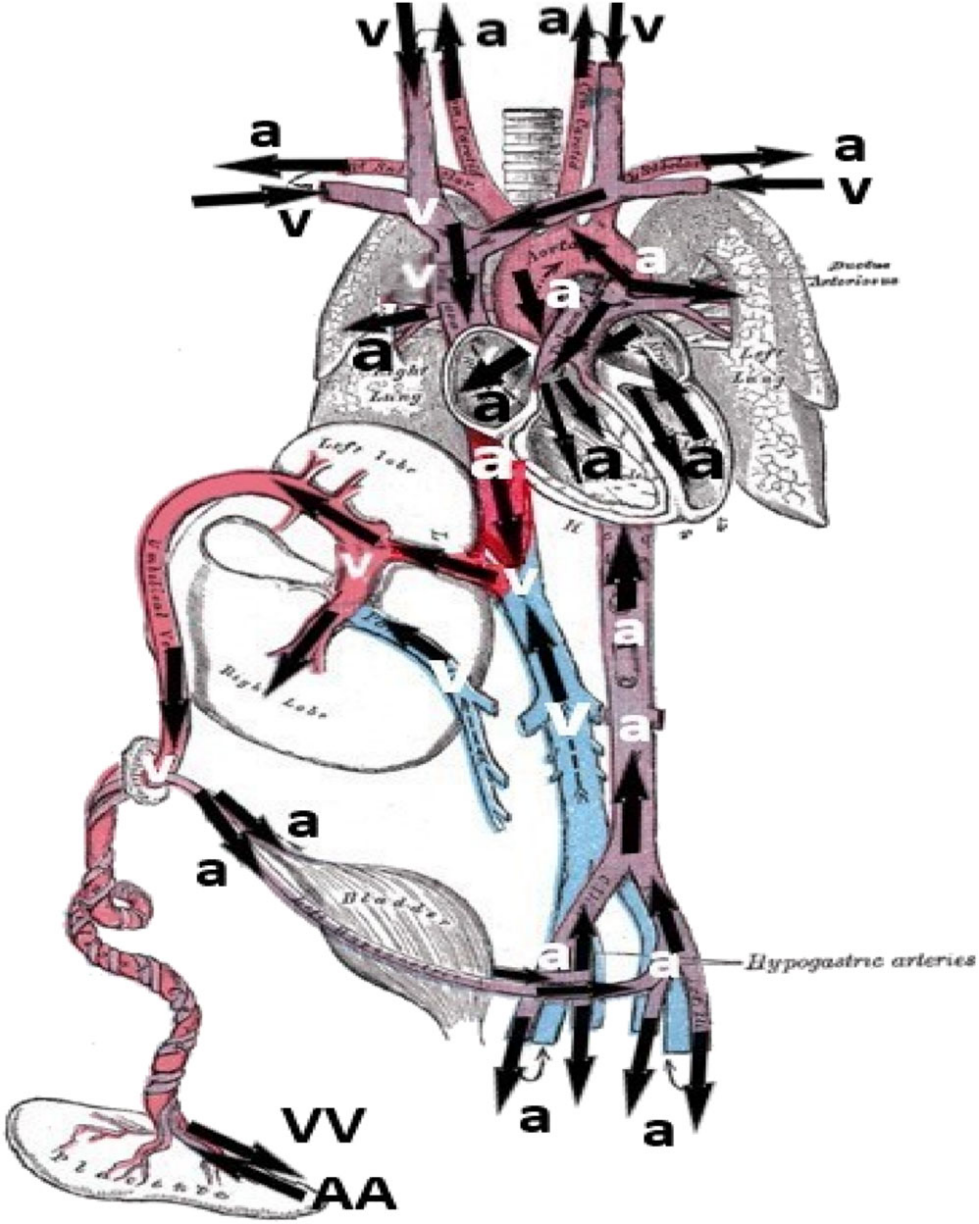
From fig. 502 of Gray (1918). Ductus venosus connecting to the inferior vena cava. Section 4c. Peculiarities in the vascular system in the fetus. We added the blood flows (black arrows) measured by Gembruch, Viski, Bagamery, Berg, and Germer (2001) in a just demised fetus at 25 + 3 weeks, during exsanguination of the other, live, twin. AA, VV are arterioarterial, venovenous; a is arterial, v is venous

The acardiac circulation determines which body parts develop normally and which body parts develop abnormally or vanish under anomalous conditions. Figure 2 shows a picture of the fetal circulation from the seminal anatomy book of Henry Gray (Gray, 1918). We adapted this picture for an acardiac twin that was just formed from a normal embryo. The directions of blood flow (black arrows, “a” is arterial, “v” is venous) is from Gembruch et al. (2001), measured at 25 + 3 weeks, 30–60 min after demise of this twin, during exsanguination of the live twin into the demised twin's circulation. We tacitly assumed that the same circulation develops in a just demised normal monochorionic twin embryo. The aortic and mitral valves were open and we therefore assumed open tricuspid and pulmonary valves too. We also assumed that the pulmonary artery and right ventricle became blood filled from the aorta through the ductus arteriosus, and the right atrium through the tricuspid valve. Retrograde arterial perfusion thus occurred in the umbilical arteries/artery, the iliac arteries, the aorta, ductus arteriosus, and the foramen ovale. Retrograde venous perfusion occurred in the ductus venosus and umbilical vein. Obviously, the extremities and head, and all parenchymal organs, except the liver, were perfused in normal arterial and venous directions.

The heart of a just formed acardius becomes filled with blood and it is unlikely that much blood will flow through the aorta, coronary arteries and heart. As a consequence, we hypothesized that this mechanism may explain why the heart of an acardiac vanishes so frequently. However, when the sinoatrial node and/or the atrioventricular node remain viable, and the heart keeps beating, a local circulation may remain present. This phenomenon of cardiac activity in an “acardiac” fetus was regularly reported in literature (e.g., van Gemert, van den Wijngaard, Paarlberg, Gardiner, & Nikkels, 2017). However, insufficient information is available on how an acardiac‐based circulation could affect the acardiac body, particularly because the criteria of reversed arterial flow for an acardiac twin pregnancy are always fulfilled. In an acardius acormus (Brand & Krol, 1941), the heart perfused the cephalic part of the acardiac at about 30 beats per minute for 1.5 hr after birth (about 8 months gestation). Most likely, this heart rate was based on the nodal rhythm of a still‐present AV node.

The liver normally receives oxygenated venous blood directly from the placenta via the umbilical vein. Most of this blood is distributed through the liver, the remaining part is directed through the ductus venosus toward the right atrium and through the foramen ovale into the left atrium and left ventricle. During acardiac development, this oxygenated venous blood flow is no longer present, which might explain that the highly metabolically active liver almost always vanishes in acardiac twins (see, e.g., Giménez‐Scherer & Davies, 2002).

The frequency of altered/disappearing organs in monochorionic twins delivered with an acardiac is not random but decreases in craniocaudal direction, a consequence of the retrograde low‐oxygenated blood perfusion through the aorta that occurred from onset to delivery Giménez‐Scherer and Davies (2002). They further reported that an absent head correlated with fewer malformed pelvic organs (19% vs. 41%), but with slightly more malformed thoracic and abdominal organs (63% vs. 56%). Also, an absent head correlated with legs that consist less often of missing bones (17% vs. 37%), although arms did not have that correlation (58% vs. 59%). We hypothesized that the diameter of the AA anastomosis, thus its vascular resistance, may play a role here. Detailed descriptions of the caliber of AA anastomoses in relation with the acardiac anatomy are not routinely described in literature to support or refute this hypothesis. Furthermore, it is not well‐known how fetal organ development and degeneration occurs in severely hypoxic/ischemic conditions.

Generally, a larger diameter AA anastomosis implies a larger retrograde flow in the preacardiac aorta, thus a lower arterial pressure in the thorax and in cranial direction, and earlier acardiac onset. The combination of lower arterial pressure and earlier acardiac onset may explain that upper limb formation, completed around 10 weeks, succeeds in about 50% of acephalus acardii. We also hypothesized that lower limb formation, which is not or less affected here, is due to the higher pelvic arterial pressures.

For smaller AA diameters, the opposite applies, thus smaller retrograde aorta flow in the preacardiac body, a higher arterial pressure in the thorax and in cranial direction, and later acardiac onset. We thus consider upper limb formation and perfusion of the cephalic part more likely too.

### 3.5 Acardiac subcutaneous edema

Most acardiac bodies show subcutaneous edema, as observed in 27/35 (≈77%) second trimester acardiac cases by Guimaraes et al. (2011). We hypothesized that this edema is a consequence of the level of hypoxemia in acardiac bodies combined with physiological high fetal capillary permeability and high fetal interstitial compliance (e.g., Druey & Greipp, 2010). In addition, the colloid osmotic pressure in the acardiac circulation is most likely extremely low due to disappearance of the liver and concomitantly absent albumin synthesis, and this might add to the increase in subcutaneous and interstitial fluid accumulation. Hypoxia, in addition, is likely the cause of vascular/endothelial damage, which further increases the accumulation of extracellular interstitial fluid.

### 3.6 Acardiac development after onset

An interesting question is whether first trimester‐formed acardiac twins may change anatomically and/or morphologically beyond the moment of onset. Despite current first trimester screening for chromosomal abnormalities (e.g., Lewi et al., 2010), this question has to our knowledge not frequently been addressed.

In a few publications, post first trimester cases were described without precise documentation of acardiac anatomy. Moore et al. (1990) found in 49 acardiac cases beyond 24 weeks that 70% had a heavier weight than their pump twins, and 25% even a 1.4 times heavier weight. In contrast, Lewi et al. (2010) found first trimester CRL of pump twins on average to be much larger than upper pole‐rump length of acardiacs, 62 ± 8 vs. 36 ± 12 mm (*p* < .001). The heavier acardiacs reported by Moore et al. (1990), therefore, could have been developed beyond the first trimester.

Van Gaever et al. (2008) showed a delayed development of lower limbs and spine following diagnosis of an acardius amorphus at 11 + 4 weeks. Another example is the acardius amorphus consisting of an intestinal sac with fluid and one leg that vanished at 34 weeks (Sharbaf et al., 2008). Also, the acardius acormus with beating heart, and numerous internal organs present inside a sac attached to the lower side of the small head, of 10 × 6 × 6 cm^3^ (Brand & Krol, 1941) must very likely have been due to anatomical changes that occurred after its first trimester onset.

Thus, acardiac morphology may change during pregnancy. For an acephalus, we predict that the various body forms, varying from full body to only a lower body (fig. 8 of Sato et al., 1984), may also have developed beyond the first trimester.

### 3.7 Single acardiac umbilical artery from Hyrtl's anastomosis

Hyrtl's anastomosis (Hyrtl, 1870) connects the two umbilical arteries a few cm above the placental cord insertion in ≈98% of cases (Ullberg, Sandstedt, & Lingman, 2001; Valsalan et al., 2018), at the flow end of the umbilical cord, just before the blood enters the placenta. In acardiacs, however, Hyrtl's anastomosis is *at the beginning* of the retrograde umbilical arterial blood flow, just next to the AA placental junction, and before the remaining umbilical arterial length toward the acardiac body. This anatomy implies that Hyrtl's anastomotic resistance affects retrograde flow in umbilical arteries much more than under normal conditions, and hence can affect the umbilical artery morphology. It is well‐known that (fetal) vessels may obliterate and become atretic with diminished flow.

**FIGURE 3:**
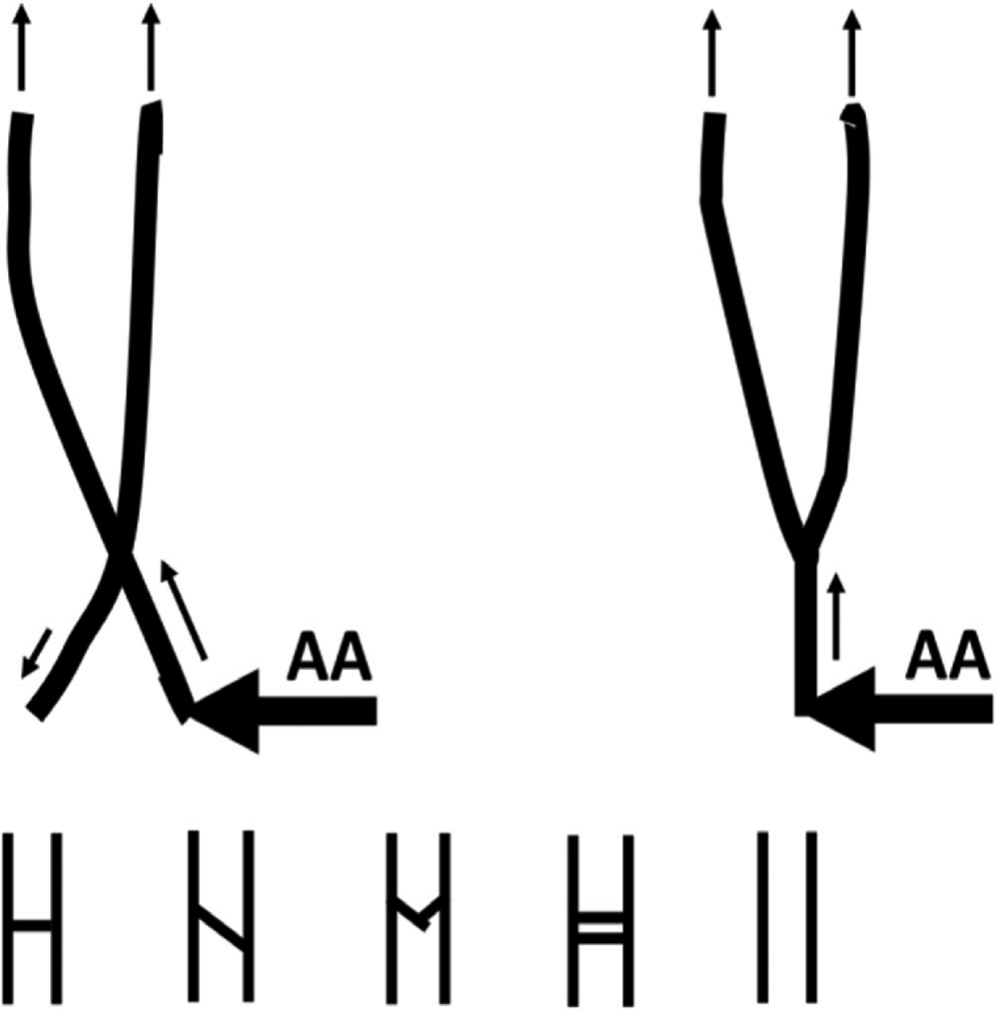
Left upper: two umbilical arteries with a fenestrated Hyrtl's anastomosis, and the AA connected to one of the umbilical arteries. Right upper: two umbilical arteries with a fused Hyrtl's anastomosis, and the AA connected to the fused umbilical artery. The retrograde directed blood flows in the umbilical arteries are indicated with small black arrows. We have assumed that fused Hyrtl's anastomoses and 50% of the fenestrated anastomoses retain two umbilical arteries. The five smaller lower figures are other Hyrtl's anastomoses from Valsalan et al. (2018) and Ullberg et al. (2001). We have assumed that each of these five lower cases will develop into one umbilical artery

Guimaraes et al. (2011) reported a single umbilical artery in 30 of 35 (86%) acardiac cases and we hypothesized that Hyrtl's anastomosis caused this anomaly. Valsalan et al. (2018), their fig. 1 and tables 1 and 2, found six types of Hyrtl's anastomoses next to agenesis of this anastomosis, in 111 pregnancies. Figure 3, upper picture, shows the AA connected to one umbilical artery in a fenestrated Hyrtl's anastomosis (left) and to a fused anastomosis (right). We hypothesized that the observed four fused and perhaps some of the 26 fenestrated Hyrtl's anastomoses will remain patent, implying that both umbilical arteries persist. We further hypothesized that only one umbilical artery develops when any of the other four Hyrtl's anastomoses are present, as well as cases without Hyrtl's anastomosis, shown in the lower part of Figure 3. Hyrtl's anastomotic resistance triggers a decreased reversed distal blood flow in the other, not AA connected, acardiac umbilical artery, which diminishes its vascular growth and eventually causing this umbilical artery to vanish. Thus, if the four fused Hyrtl’s anastomoses remain patent, and including the two cases without Hyrtl's anastomosis, one umbilical artery would develop in 111 − (4 + 2) = 105 or in ≈105/111 ≈95% of cases. If, say, 50% of the 26 fenestrated Hyrtl's anastomoses also remain patent, ≈83% single umbilical arteries would develop. Furthermore, Ullberg et al. (2001) found similar anatomies in 67 Hyrtl's anastomoses. Using again that the one fused and 50% of the four fenestrated anastomoses remain patent, and including the two cases without Hyrtl's anastomosis, one umbilical artery would develop in 67 − (5 + 2) = 60 cases, or in about 90%.

Thus, combining Hyrtl's anastomotic resistance‐related reduction of retrograde umbilical arterial flow when an AA connects to one umbilical artery only, predicts a process of obliteration and subsequent disappearance of the other umbilical artery, and quite well the observed large incidence, ≈86%, of a single umbilical artery seen in acardiac twins.

### 3.8 Risk prediction of pump twins by UVD ratios

**FIGURE 4:**
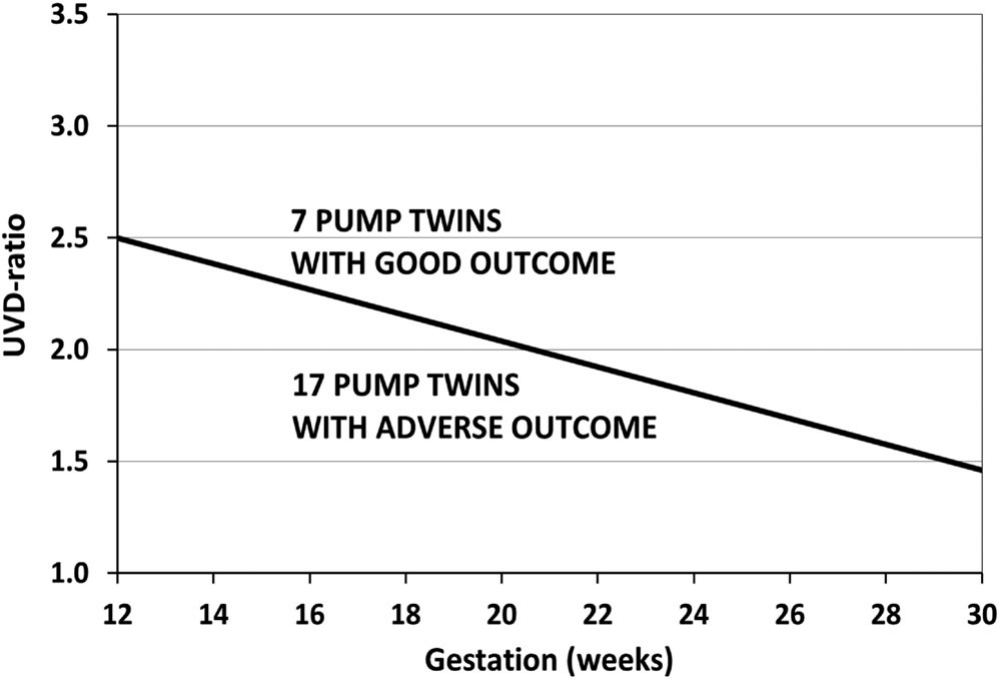
Pump/Acardiac umbilical venous diameter (UVD) ratios separating 17 pump twins with adverse outcome from 7 without adverse outcome (from fig. 4 of van Gemert et al., 2016). The separation line is drawn by eye and has no scientific foundation

The classification of acardiacs into four morphological body appearances is well‐known to have no relation with the risk of pump twins (e.g., Wong & Sepulveda, 2005). We published a very simple clinical method (van Gemert et al., 2016), based on pump/acardiac UVD ratios, that provides pump twin risk of cardiac overload, Appendix A, Equation (1). In Figure 4, we provided a line of UVD ratios versus gestation that separated seven acardiac cases without pump twin complications from 17 cases with adverse pump twin outcome.

## 4 DISCUSSION

### 4.1 Summary of findings and literature perspectives

A summary of our results is given in Table 5.

**TABLE 5:** Summary of findings

Acardius	Incidence (Table 2)	Predicted incidence	Predicted onset	Spontan. abortions	Normal onset	Changes postonset	AA diam.	Comment
Acephalus	≈64%	?	<10 weeks	Perhaps	Yes	Likely	Larger	Cranial‐caudal altered organs; also in first trimester?
Amorphus	≈22%	≈23%	Early	Yes	Perhaps	?	?	Predicted incidence from spontaneous abortions
Anceps	≈10%	?	>10 weeks	Perhaps	Yes	Likely	Smaller	Smaller than acephalus?
Acormus	≈4%	>2.5%	>10 weeks	Likely	Yes	Likely	Larger	SUC
Single UA	≈86%	83%–95%	?	NA	NA	Possible? (2UA to 1UA)	NA	Hyrtl's anastomosis
Edematous	≈77%	?	?	NA	NA	Likely	NA	Hypoxia‐linked capillary leakage
With SUC	?	>1.3%	First trimester	Likely	Yes	Likely	NA	Novel acardiac etiology

Abbreviations: NA, not applicable; SUC, short umbilical cord; UA, umbilical artery.

We predicted that an acardius acephalus develops prior to 10 weeks and requires a larger diameter AA anastomosis than onset of an anceps, which develops after 10 weeks, after bone formation of the skull. Acephalus onset before 10 weeks was observed by Coulam (1996) and Wesley et al. (2011), and a large size AA anastomosis by Wesley et al. (2011). Stiller et al. (1989) and Meyberg and Groß (2002) confirmed our anceps prediction (observed at, respectively, ≈11 and 10 + 6 weeks). However, Masuzaki, Miura, Yoshimura, Yoshiura, and Ishimaru (2004) observed an acardius anceps with cranial bones in a partially developed head and face and upper and lower left limbs at already 9 weeks, thus prior to completion of the cranium at 10 weeks. This observation may show that organ formation such as cranial bones can also continue during acardiac retrograde perfusion. In other words, acardiac onset may not strictly follow normal organogenesis.

We hypothesized that first trimester acaphalus could possibly be larger than anceps acardii because the AA flow perfuses the acardiac body, causing its growth, and the AA flow in earlier developing acephalus acardii exceeds that of anceps acardii (Section 3.1). If true, also the UVD ratio of acephalus exceeds that of anceps acardii, a prediction that can easily be verified.

We are the first to provide a mechanism for single umbilical artery development in acardiacs, based on the various anatomies observed of Hyrtl's anastomosis (Section 3.7). We included that the reversed perfused umbilical artery that is not directly connected to the AA can vanish distal to Hyrtl's anastomosis based on reduced retrograde flow in that direction due to the anastomotic resistance. Based on types and incidences of Hyrtl's anastomoses (Ullberg et al., 2001; Valsalan et al., 2018), the large incidence of a single anastomosis, ≈86%, could be reasonably well‐predicted. Interesting is whether a single umbilical artery develops before or after acardiac onset.

We also suggested that the physiological high fetal capillary permeability combined with high compliance of the interstitium with hypoxemia cause the formation of an edematous acardiac body (Section 3.5). Furthermore (Section 3.4), we suggested an explanation for the observation that the liver is almost always absent in acardiac fetuses. During acardiac development, the highly oxygenated venous blood flow from the placenta and umbilical vein is no longer present which may result in disappearance of the highly metabolically active liver.

Also novel is the suggestion (Section 3.3) that acardiac twinning will result if one of the monochorionic twin embryos has a very short umbilical cord, assumed to occur in almost 10% (vs. 6% in singletons), which can occur before skull formation, <10 weeks, as well as >10 weeks. The latter case is the hypothesized pathogenesis of acardius acormus.

Obviously, the four body morphologies may be mixed. Examples are acardius amorphus with rudimentary limb formation (Boronow & West, 1964; Sato et al., 1984), a beating heart (Alves, Brasileiro, de Aquino Campos, & Pitombeira Ferreira, 1998), and rudiments of thoracic structures (Bonilla‐Musoles, Machado, Raga, & Osborne, 2001). Furthermore, an acardius acormus with a rudimentary body (Abi‐Nader, Whitten, Filippi, Scott, & Jauniaux, 2009; Sato et al., 1984), a protruding brain (Morizane et al., 2002), and mixed features with an acardius amorphus (Huss et al., 2011; Martínas‐Frías, 2009, fig. 3, case 8). A fifth morphology, acardius mylacephalus (Table 2), was proposed as an amorphous mass with some development of one or more limbs, for example, by Emery et al. (2004). This morphology is often attributed to Simmonds and Gowen (1925), but Das (1902) mentioned the British Anatomist‐Pathologist‐Surgeon Alban Henry Griffith Doran (1849‐1927) who introduced this name but who later regrouped such cases as acephalus. Neitzschman, Jacobs, Genet, and Nice (1973) described an acardius acormus, which in our opinion should be named acardius anceps due to the presence of a single lower extremity.

Interestingly, Schatz (1898) suggested that an omphalocele was perhaps etiologic in acardiac twinning because it caused obstruction to venous return (from Emery et al., 2004). We submit that the etiology of acardiac twinning by a short umbilical cord at 6–10 weeks, when an omphalocele is present, is currently the closest mechanism to Schatz's suggestion.

Acardiac twins have also been described in dichorionic fused placentas, by Jirásek (reported in Schinzel, Smith, & Miller, 1979), Gewolb, Freedman, Kleinman, and Hobbins (1983), French et al. (1998), and Kline et al. (2002). Finally, Shih et al. (1999) identified a set of AV–VA anastomoses instead of AA–VV. Despite that this anatomy provides preferential blood flow to go to the upper body, the arms were more deformed than the legs. Therefore, more likely is that AA–VV anastomoses existed but thrombosed, for unknown reasons. Support comes from the much larger vascular resistance of AV–VA anastomoses than an AA with equal diameter (appendix of Umur, van Gemert, Nikkels, & Ross, 2002).

Finally, we submit that the results of Giménez‐Scherer and Davies (2002), showing that acardiacs after delivery have altered/disappearing organs with a nonrandom but decreasing frequency in craniocaudal direction, Section 3.4, are very likely from morphology changes after acardiac formation, Section 3.6. It would be interesting to confirm or refute these findings in the first trimester.

### 4.2 Limitations of the study

First, the major limitation of our study is the current lack of knowledge of abnormal organ development and degeneration under conditions of hypoxemia. This limitation prevented us from creating a computer model that could have explained the pathophysiology of this unique obstetric complication. It requires at least quantitative knowledge on how organs will vanish under “normal” conditions, by apoptosis, necrosis and probably also in part by autophagy (e.g., Robbins & Cotran's Pathologic Basis of Disease, i.e., Kumar, Abbas, & Aster, 2020). The process of necrosis is probably less likely in the embryo. It causes an inflammatory reaction with destruction of surrounding tissue with subsequent degeneration and calcification. This is in most cases not observed and apoptosis and autophagy are more likely to occur at this very early age.

Second, another important limitation is the lack of data of early monochorionic twin pregnancies. Significant is our assumption that singleton spontaneous aborted embryos (Poland et al., 1981) may possibly also be representative for frequency and morphology of aborted monochorionic embryos with a surviving coembryo. Obviously, this challenging assumption can currently neither be confirmed nor refuted. Nevertheless, it was helpful in estimating the incidences of acardii amorphus and acormus. For acardius amorphus (Section 3.2), it showed a possible and unexpected cause of its relatively large incidence, which was in our view against expectation. Equally so, it provided at least some clues for the small incidence of acormus and other short umbilical cord acardii (Section 3.3). Furthermore, spontaneous abortion statistics played no role in acardius acephalus and anceps formation, Section 3.1.

Third, Poland et al. (1981) reported in their table XI that 129 of 228 spontaneous aborted singleton embryos (57%) had abnormal karyotyping; that 87 of 99 normal karyotyped embryos were abnormal, and that 16 normal embryos included 12 with normal karyotyping. Soler et al. (2017) found a 70% incidence of chromosomal anomaly in 1,011 first trimester miscarriages. However, Philipp et al. (2003) observed even a 75% abnormal chromosomal incidence in 221 spontaneous singleton abortions. Nevertheless, we did not discuss acardiacs with abnormal karyotyping in detail, despite published examples, for example, Moore et al. (1987), and Søgaard et al. (1999), because such spontaneous abortion specimens of singleton pregnancies showed no significant differences in severity and morphology from normal karyotyped species (discussion of Philipp et al., 2003).

### 4.3 Suggestions for further study

An important area of study would be ultrasound examination of acardiac twins, including the period between pre‐ and postonset, and assessment of morphology, for example, according to Gembruch et al. (2001). This would provide information during pregnancy of organ formation similar to what Giménez‐Scherer and Davies (2002) determined after delivery. Such information could provide knowledge on how organs behave during perfusion with low‐oxygenated blood.

A second major area is to combine the above mentioned information with UVD ratios (Appendix A). UVD ratios can also assess the oxygen saturation of the AA blood flow (van Gemert et al., 2016), and therefore in concept also of acardiac arteries and veins (Appendix A, Equations 5 and 6). Then, using a (future) acardiac circulatory model, for example, analogous to previous work (van den Wijngaard et al., 2006), and including oxygenation values, could result in the much wanted analysis of acardiac pathophysiology, from the preacardiac period through onset and acardiac progression. Such an approach could then resolve our first mentioned major limitation.

### 4.4 Conclusion

This study, to our knowledge the first where extensive acardiac scientific and clinical information was combined, generated hypotheses on mechanisms of acardiac onset, morphology, incidence and progression, and showed reasonable agreement with clinical observations. Novel concepts were included. First, acardiac amorphous formation relates strongly to the large incidence of amorphous spontaneous abortions. Second, acardiac formation as a result of the short umbilical cord syndrome, including acardius acormus (Section 3.3). Third, a single umbilical artery from the various anatomies of Hyrtl's anastomosis (Section 3.7), and an edematous acardiac body from physiological high fetal capillary permeability combined with high compliance of the interstitium with hypoxemia (Section 3.5). Acardiac morphology development occurring after onset was shown to be likely possible (Section 3.6). We argued that lack of knowledge on organ development and degeneration under hypoxic conditions currently prevents the design of a computer model that predicts acardiac pathogenesis and progression. However, we suggested that study of anatomy and circulation, beginning preacardiac, around onset, and continuing thereafter, like Gembruch et al. (2001), combined with oxygen saturation analysis from UVD ratios (Appendix A), and included in a future model of the acardiac circulation, could provide such a model.

Finally, many questions have yet to be answered, but we hope that our paper will stimulate to guide future studies toward acardiac twinning pathogenesis in more depth and better focused than was hitherto possible.

## CONFLICT OF INTERESTS

The authors declare no conflict of interests.

## DATA SHARING STATEMENTS

Data sharing is not applicable to this article as no datasets were generated or analyzed during the current study.

## Data Availability

Data sharing is not applicable to this article as no datasets were generated or analyzed during the current study in the system.

## Appendix A. UMBILICAL VENOUS DIAMETER RATIO

The Pump/Acardiac umbilical venous diameter (UVD) ratio relates to the pump twin's excess cardiac output, which equals the acardiac perfusion flow, as well as the blood oxygen saturation, SO_2_, in the AA anastomosis and thus in the acardiac aorta, and also in the acardiac veins. The following relations are based on the assumptions that the placenta is perfused by 30% of the fetal cardiac output, that normal oxygen saturation of an umbilical vein is 0.8 and that fetal perfusion reduces that value by 0.15, thus occurring in the umbilical arteries, and also a reduction of 0.15 in case of acardiac body perfusion. As previously, we used that the UVD ratio to the third power is the ratio of the two umbilical venous flows. Thus (van Gemert et al., 2005),

(1)
$$\text{Pump excess cardiac output}\approx \frac{2\times 0.15}{{\left(\mathrm{UVD}\;\text{ratio}\right)}^{3}-1}$$

(2)
$${\mathrm{SO}}_{2}\left(\mathrm{AA}\right)\approx 0.65-\frac{2\times 0.15}{{\left(\mathrm{UVD}\;\text{ratio}\right)}^{3}-1}$$

(3)
$${\mathrm{SO}}_{2}\left(\text{Acardiac veins}\right)\approx 0.5-\frac{2\times 0.15}{{\left(\mathrm{UVD}\;\text{ratio}\right)}^{3}-1}$$

Importantly, these relations do not apply in the first trimester due to the pregnancy's low oxygen tension, the normal situation here. Jauniaux et al. (2000) measured oxygen pressure values in first trimester placental villi of about 20 mmHg (SO_2_ ≈ 30%) at 60 gestational days, about 26 mmHg (SO_2_ ≈ 40%) at 70 days, and up to 50 mmHg from 85 days onward (SO_2_ ≈ 80%). The embryo's first trimester arterial SO_2_ values are unknown but likely not much smaller than the venous values (Huppertz, Weiss, & Moser, 2014). Then, calling the difference between venous and arterial SO_2_ values $\Delta {\mathrm{SO}}_{2}$, and using ${\mathrm{SO}}_{2}\approx 0.3$, Equations ((1), (2), (3)) become

(4)
$$\text{Pump excess cardiac output}=\frac{2\times \Delta {\mathrm{SO}}_{2}}{{\left(\mathrm{UVD}\;\text{ratio}\right)}^{3}-1}$$

(5)
$${\mathrm{SO}}_{2}\left(\mathrm{AA}\right)\approx 0.3-\frac{2\times \Delta {\mathrm{SO}}_{2}}{{\left(\mathrm{UVD}\;\text{ratio}\right)}^{3}-1}$$

(6)
$${\mathrm{SO}}_{2}\left(\mathrm{Ac}\;\text{veins}\right)\approx \left(0.3-\Delta {\mathrm{SO}}_{2}\right)-\frac{2\times \Delta {\mathrm{SO}}_{2}}{{\left(\mathrm{UVD}\;\text{ratio}\right)}^{3}-1}$$

Using these relations quantitatively obviously requires an estimate of $\Delta {\mathrm{SO}}_{2}$.
